# *LMNA* Reduced Acquired Resistance to Erlotinib in NSCLC by Reversing the Epithelial–Mesenchymal Transition via the FGFR/MAPK/c-fos Signaling Pathway

**DOI:** 10.3390/ijms232113237

**Published:** 2022-10-31

**Authors:** Chunsheng Hu, Anting Zhou, Xin Hu, Yu Xiang, Mengjun Huang, Jiuhong Huang, Donglin Yang, Yan Tang

**Affiliations:** 1 College of Pharmacy (International Academy of Targeted Therapeutics and Innovation), Chongqing University of Arts and Sciences, Chongqing 402160, China; 2 School of Life Sciences, Chongqing University, Chongqing 401331, China; 3 National & Local Joint Engineering Research Center of Targeted and Innovative Therapeutics, Chongqing University of Arts and Sciences, Chongqing 402160, China

**Keywords:** *LMNA*, epithelial–mesenchymal transition, EGFR-TKI resistance, non-small-cell lung cancer

## Abstract

For patients exhibiting non-small-cell lung cancer (NSCLC) with activating epidermal growth factor receptor (EGFR) mutations, epidermal growth factor receptor tyrosine kinase inhibitors (EGFR-TKIs) are a first-line treatment. However, most patients who initially responded to EGFR-TKIs eventually developed acquired resistance, limiting the effectiveness of therapy. It has long been known that epithelial–mesenchymal transition (EMT) leads to acquired resistance to EGFR-TKIs in NSCLC. However, the mechanisms underlying the resistance dependent on EMT are unknown. This research aimed to reveal the effects of *LMNA* in the regulation of acquired resistance to erlotinib by EMT in NSCLC. The acquired erlotinib-resistant cells (HCC827/ER) were induced by gradual increase of concentrations of erlotinib in erlotinib-sensitive HCC827 cells. RNA sequencing and bioinformatics analysis were performed to uncover the involvement of *LMNA* in the EMT process that induced acquired resistance to erlotinib. The effect of *LMNA* on cell proliferation and migration was measured by clone-formation, wound-healing, and transwell assays, respectively. The EMT-related protein, nuclear shape and volume, and cytoskeleton changes were examined by immunofluorescence. Western blot was used to identify the underlying molecular mechanism of *LMNA* regulation of EMT. HCC827/ER cells with acquired resistance to erlotinib underwent EMT and exhibited lower *LMNA* expression compared to parental sensitive cells. *LMNA* negatively regulated the expression of EMT markers; HCC827/ER cells showed a significant up-regulation of mesenchymal markers, such as *CDH2*, *SNAI2*, *VIM*, *ZEB1*, and *TWIST1*. The overexpression of *LMNA* in HCC827/ER cells significantly inhibited EMT and cell proliferation, and this inhibitory effect of *LMNA* was enhanced in the presence of 2.5 μM erlotinib. Furthermore, a decrease in *LMNA* expression resulted in a higher nuclear deformability and cytoskeletal changes. In HCC827/ER cells, AKT, FGFR, ERK1/2, and c-fos phosphorylation levels were higher than those in HCC827 cells; Furthermore, overexpression of *LMNA* in HCC827/ER cells reduced the phosphorylation of AKT, ERK1/2, c-fos, and FGFR. In conclusion, our findings first demonstrated that downregulation of *LMNA* promotes acquired EGFR-TKI resistance in NSCLC with EGFR mutations by EMT. *LMNA* inhibits cell proliferation and migration of erlotinib-resistant cells via inhibition of the FGFR/MAPK/c-fos signaling pathway. These findings indicated *LMNA* as a driver of acquired resistance to erlotinib and provided important information about the development of resistance to erlotinib treatment in NSCLC patients with EGFR mutations.

## 1. Introduction

Lung cancer is one of the most malignant tumors, with a high lethality rate in the world. It comprises two different groups: small-cell lung cancer (SCLC) and non-small-cell lung cancer (NSCLC). Among NSCLC, lung adenocarcinoma represents the major histological subtype, accounting for 60% of NSCLC [1]. In recent years, epidermal growth factor receptor (EGFR) tyrosine kinase inhibitors (TKIs), such as gefitinib, erlotinib, and afatinib, have been approved to treat patients with NSCLC that harbors an activating mutation of EGFR [2]. However, the vast majority of patients developed drug resistance within 1 to 2 years of treatment [3]. Several mechanisms of resistance to EGFR-TKIs have been identified, such as secondary mutation (T790M) of EGFR, activation of alternative signaling pathways (c-MET, HGF, AXL, HER2), loss of PTEN, histological transformation, etc. [4,5] However, other mechanisms expected to be involved in resistance to EGFR-TKIs are still poorly understood. Therefore, elucidation of new mechanisms is important to avoid drug resistance and to develop novel therapeutic strategies of treating patients with NSCLC.

Nuclear lamins are one of the main components of the nuclear matrix, including the nuclear lamina, which is a network of lamin filaments located in the interior of the nuclear envelope [6]. Lamins are divided into A- and B-types. Two A-type lamin isoforms (lamin A and C, called laminA/C) are produced through alternative *LMNA* splice variants [7]. Previous studies have suggested that laminA/C are involved in the development and progression of tumors [8,9,10,11]. LaminA/C deficiency caused a decrease in nuclear periphery and an alteration of nuclear shape [12]. Furthermore, the epithelial-to-mesenchymal transition (EMT) in cancer cells was associated with a decrease in laminA/C expression [13,14]. Previously, studies had suggested that A-type lamins play an important role in the development and prognosis of human carcinoma [15,16,17]. However, how laminA/C mediates the acquired resistance to EGFR-TKIs in NSCLC remained elusive.

In the present study, we investigated the effects of *LMNA* on acquired resistance to erlotinib in lung cancer cells and studied the underlying molecular mechanisms involved. Knockdown of *LMNA* significantly reduced erlotinib sensibility in HCC827 by phenotypic transformation of EMT. Furthermore, overexpression of *LMNA* significantly reversed resistance to erlotinib in HCC827/ER erlotinib-resistant cells by reversing EMT. Our findings provided a potential target to reverse EGFR-TKIs resistance.

## 2. Results

### 2.1. Global Transcriptome Changes between Parental and Erlotinib-Resistant Cells

Acquired resistance to erlotinib was induced in the erlotininb-sensitive HCC827 cell line by gradual increase of concentrations of erlotinib ranging from 0.005 to 2.5 μM. Erlotinib-resistant cells (HCC827/ER) were established after 6 months with erlotinib exposure, and the cells no longer responded to erlotinib concentrations up to 5 μM. To evaluate differential expressions of genes (DEGs) and underlying mechanisms of resistance to erlotinib between the HCC827 and HCC827/ER cell lines, we sequenced HCC827 and HCC827/ER cells using the BGISEQ-500 platform, which generated an average of approximately 24.02 M reads per sample.

**Figure 1:**
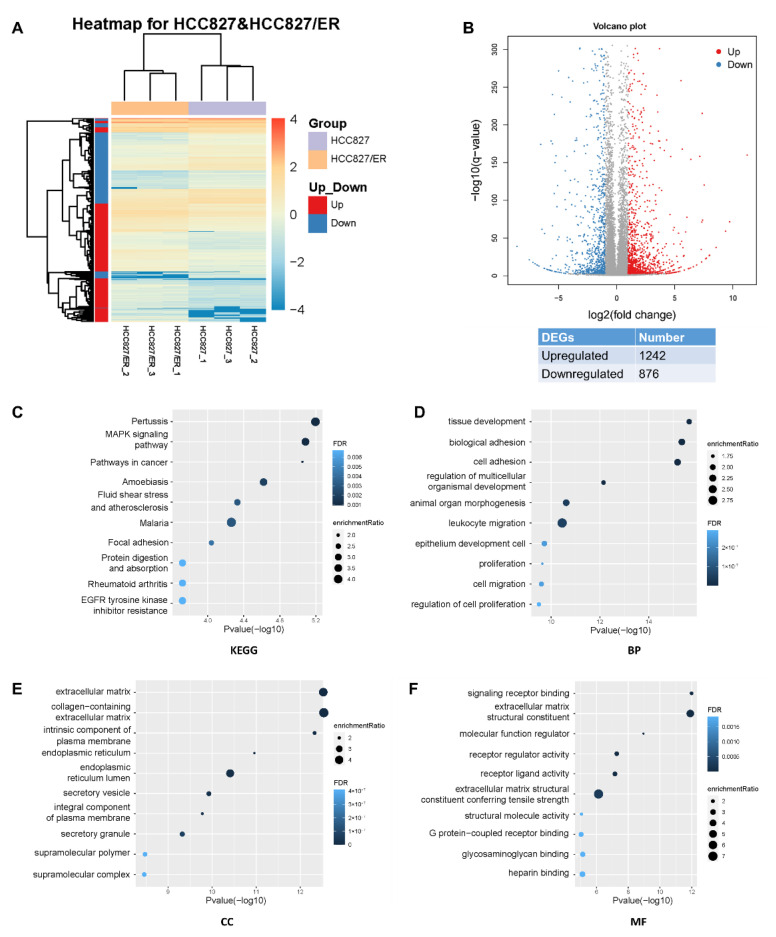
Profound changes in the global transcriptional landscape between parental and erlotinib-resistant cells. (**A**) Heatmap of genes that were differentially expressed (DEGs) between HCC827 and HCC827/ER cells. The color represents the level of transformed gene expression of log10. (**B**) Volcano plot displaying genes detected by RNA-Seq. Red points represent up-regulated DEGs (FoldChange ≥ 2, q value ≤ 0.001). Blue points represent down-regulated DEGs (FoldChange ≤ −2, q value ≤ 0.001). Gray points represent non-DEGs. (**C**) KEGG pathway enrichment analysis of genes up-regulated or down-regulated in pairwise comparison of HCC827 and HCC827/ER cells. FDR ˂ 0.05 signified statistical significance. (**D**) Biological process, (**E**) cellular components, and (**F**) molecular function annotations of gene ontology were significantly enriched in genes that were differentially expressed between HCC827 and HCC827/ER cells. Top 10 terms of each class were performed. FDR ˂ 0.05 signified statistical significance.

Analysis of RNA-seq data identified 2118 DEGs (*p* ≤ 0.05, *q* ≤ 0.05, |fold change| ≥ 2), of which 1242 and 876 were up- and down-regulated, respectively (Figure 1A,B). In the heat map of the DEGs, three HCC827 and three HCC827/ER samples were clearly separated by hierarchical clustering, demonstrating robust transcriptome changes after resistance to erlotinib. From a further analysis of Kyoto Encyclopedia of Genes and Genomes (KEGG) pathways, we found that the most enriched pathways, such as MAPK, pathways in cancer, adhesion, and EGFR tyrosine kinase inhibitor resistance pathways, affected cell migration and proliferation (Figure 1C). Furthermore, gene ontology analysis showed that erlotinib resistance affected biological processes (BP), cellular components (CC), and molecular functions (MF) by regulating 2118 genes in various pathways (Figure 1D–F), such as cell adhesion, cell migration, and extracellular matrix. Together, these results suggested that EMT is associated with acquired resistance of lung adenocarcinoma to erlotinib.

### 2.2. Expression Analysis of LMNA and EMT Markers in HCC827 and HCC827/ER Cells

**Figure 2:**
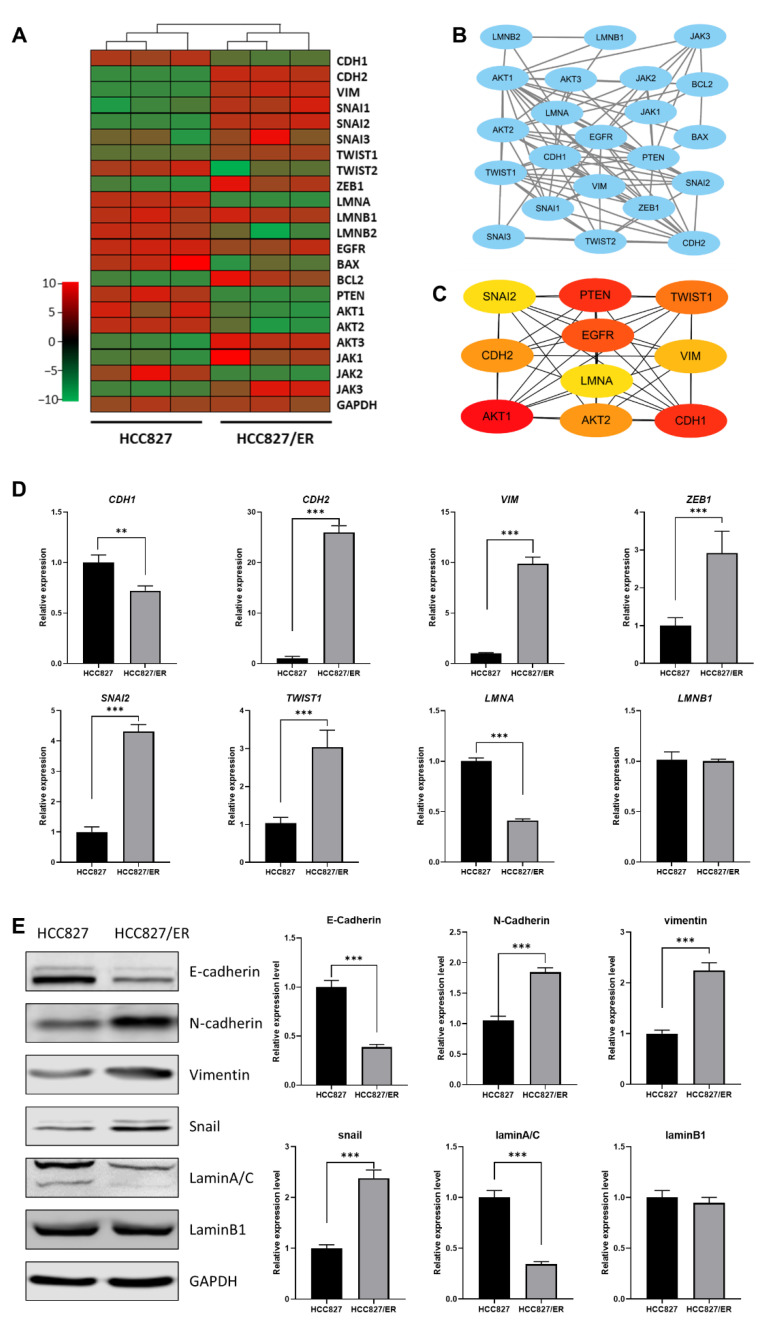
Relationship between *LMNA* and the EMT-related gene between HCC827 and HCC827/ER cells. (**A**) Heatmap illustrating the expression of genes related to *LMNA* and EMT between HCC827 and HCC827/ER cells. (**B**) An interaction network using the STRING online database and Cytoscape software for 22 common gene signatures involved in resistance to erlotinib and EMT mechanisms. (**C**) A total of 10 genes were identified as hub genes from 22 candidate genes using the CytoHubba tool in Cytoscape: *LMNA*, *EGFR*, *PTEN*, *CDH1*, *CDH2*, *VIM*, *SNAI2*, *TWIST1*, *AKT1*, and *AKT2*. (**D**) Relative expression levels of genes related to *LMNA* and EMT in HCC827 and HCC827/ER cells were confirmed by qPCR. Data are presented as mean ± SD. ** *p*< 0.001, *** *p* < 0.001. (**E**) The expression of laminA/C, laminB1, E-cadherin, N-cadherin, vimentin, and snail was analyzed by Western blot in HCC827 and HCC827/ER cells. GAPDH was used as a loading control.

To more deeply explore the potential relationship between *LMNA* and EMT in acquired resistance to erlotinib, we analyzed the correlation between expression of *LMNA* and EMT markers and constructed an interaction network between *LMNA* and related genes by predicting a STRING network. DEG RNA sequencing analysis illustrated that *LMNA* and *CDH1* (an epithelial marker) were significantly down-regulated in HCC827/ER cells. However, mesenchymal markers, such as *CDH2*, *SNAI2*, *VIM*, *ZEB1*, and *TWIST1*, were significantly up-regulated in HCC827/ER cells (Figure 2A). Furthermore, *LMNA* and some key genes involved in EMT signaling were integrated into the protein–protein interaction network using the STRING database (Figure 2B). In addition, 10 DEGs were identified as hub genes using cytoHubba in Cytoscape software, and a connected module of the hub genes was obtained by Cytoscape with MCC (Figure 2C). These results suggested that *LMNA* could regulate MAPK signaling and the expression of EMT-related genes. Moreover, to validate RNA-seq data, we selected several DEGs for qRT-PCR and Western blot. Expression levels calculated using RNA-seq were significantly positively correlated with expression levels determined by qRT-PCR and protein analysis (Figure 2D, E). The expression of *LMNA* (laminA/C) was down-regulated in HCC827/ER cells. The expression level of the epithelial marker E-cadherin (*CDH1*) was also lower in HCC827/ER cells as compared to HCC827 cells. On the contrary, the expression of mesenchymal markers N-cadherin (*CDH2*), vimentin (*VIM*), snail (*SNAI2*), and ZEB1 (*ZEB1*) were up-regulated in HCC827/ER cells. Together, these results demonstrated that erlotinib-resistant cells (HCC827/ER) were poorly expressed in *LMNA* and exhibited EMT features.

### 2.3. Downregulated LMNA Associated with Acquired Resistance to Erlotinib in HCC827 Cell Lines

**Figure 3:**
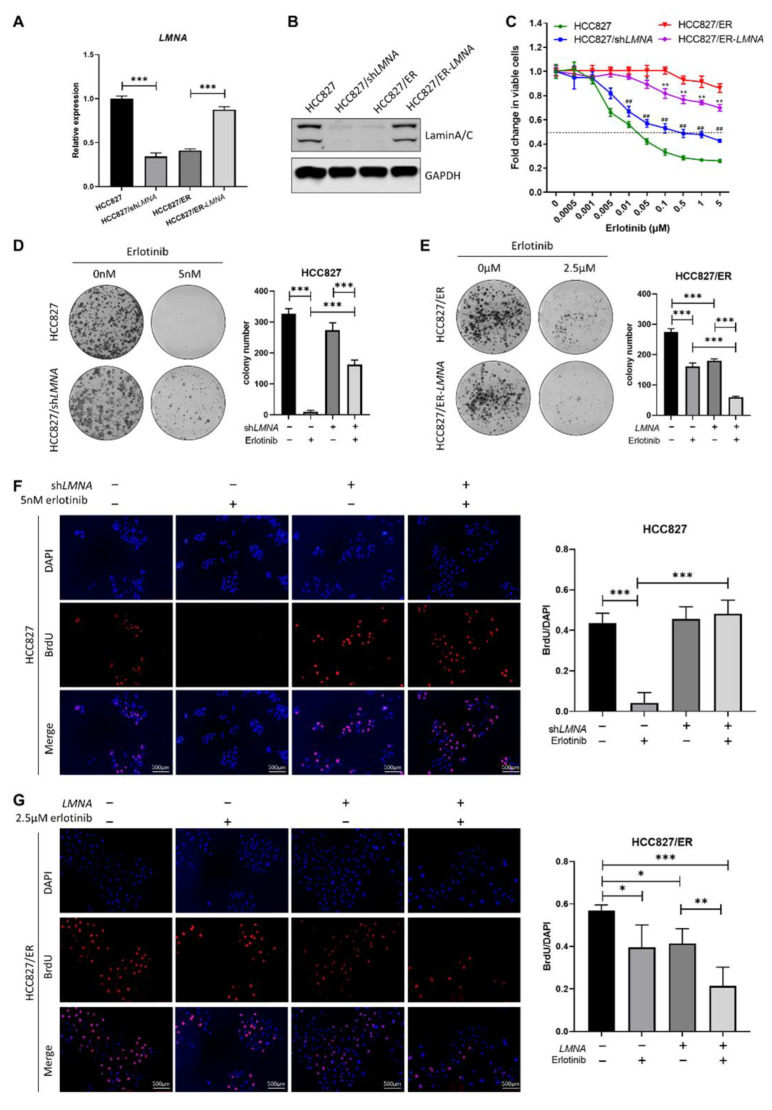
Effect of *LMNA* on cell proliferation and resistance to erlotinib in HCC827 and HCC827/ER cells. (**A**) The mRNA levels and (**B**) protein expression of *LMNA* were determined by qRT-PCR and Western blot after lentiviral infections of HCC827 cells with shRNA and HCC827/ER cells with *LMNA*, respectively. (**C**) Effect of *LMNA* knockdown or overexpression on erlotinib efficacy was determined by MTT assay. Data are presented mean ± SD from three independent experiments: ** *p* < 0.01 versus HCC827/ER cells, ## *p* < 0.01 versus HCC827 cells. (**D**, **E**) A colony-formation assay was performed to investigate the antiproliferation of *LMNA* in combination with indicated erlotinib in parental and resistant cells. Data are presented as mean ± SD from three independent experiments. Significant differences are indicated as follows: Student’s *t*-test, *** *p* < 0.001. (**F**) and (**G**) A BrdU incorporation assay was performed in HCC827 and HCC827/ER cells to assess the antiproliferative effect of *LMNA* combined with 5 nM or 2.5 μM erlotinib after 24 h of incubation, respectively. Data are presented as mean ± SD from three independent experiments. Significant differences are indicated as follows: Student’s *t*-test, * *p* < 0.05, ** *p* < 0.01, *** *p* < 0.001.

In order to explore the relationship between *LMNA* and acquired resistance to erlotinib, HCC827 cells were transfected with *LMNA* shRNA lentivirus to obtain HCC827/sh*LMNA* cells, and HCC827/ER cells were transfected with a *LMNA* overexpressing lentivirus to obtain HCC827/ER-*LMNA* cells. First, levels of *LMNA* mRNA and protein expression were detected by qRT-PCR and Western blot in the four indicated cell lines, respectively. As shown in Figure 3A, B, both *LMNA* mRNA and protein expression levels were significantly reduced in HCC827/sh*LMNA* cells as compared to those of HCC827 cells. During the mean time, the levels of mRNA and protein expression of *LMNA* increased significantly in HCC827/ER cells after transfection of the *LMNA* overexpression lentivirus. Subsequently, sensitivity of erlotinib in the four indicated cell lines was determined by MTT assay. The result showed that HCC827/sh*LMNA* cells significantly increased resistance to erlotinib as compared to HCC827 cells. On the contrary, the overexpression of lamin A/C in HCC827/ER-*LMNA* cells reversed resistance to erlotinib (Figure 3C). A clone-formation assay showed that lamin A/C removal did not affect the number of colonies in HCC827 without erlotinib, but significantly increased the size of the colonies. Furthermore, the number of colonies of HCC827/sh*LMNA* cells increased significantly compared to that of HCC827 cells in the presence of 5 nM erlotinib (Figure 3D). This situation was the opposite in HCC827/ER cell transfection with *LMNA* genes; the number of colonies decreased significantly in HCC827/ER-*LMNA* cells. Specifically, the number of colonies decreased more in HCC827/ER-*LMNA* cells treated with 2.5 μM erlotinib (Figure 3E). A BrdU assay was performed to determine whether colony formation inhibition was associated with *LMNA* that regulated cell proliferation. The results indicated that cell proliferation of HCC827 and HCC827/sh*LMNA* cells did not have any effect when no erlotinib was present. With 5 nM erlotinib, HCC827 cell proliferation was inhibited; however, there was no reduction in cell proliferation in HCC827/sh*LMNA* cells (Figure 3F). In HCC827/ER cells, *LMNA* overexpression significantly inhibited cell proliferation, and this inhibitory effect of *LMNA* was enhanced in the presence of 2.5 μM erlotinib (Figure 3G). These results suggested that *LMNA* is a crucial regulatory molecule involved in modulation of acquired resistance to erlotinib in NSCLC.

### 2.4. LMNA Prevention of Migration and Invasion of Lung Adenocarcinoma Cells’ Resistance to Erlotinib In Vitro

**Figure 4:**
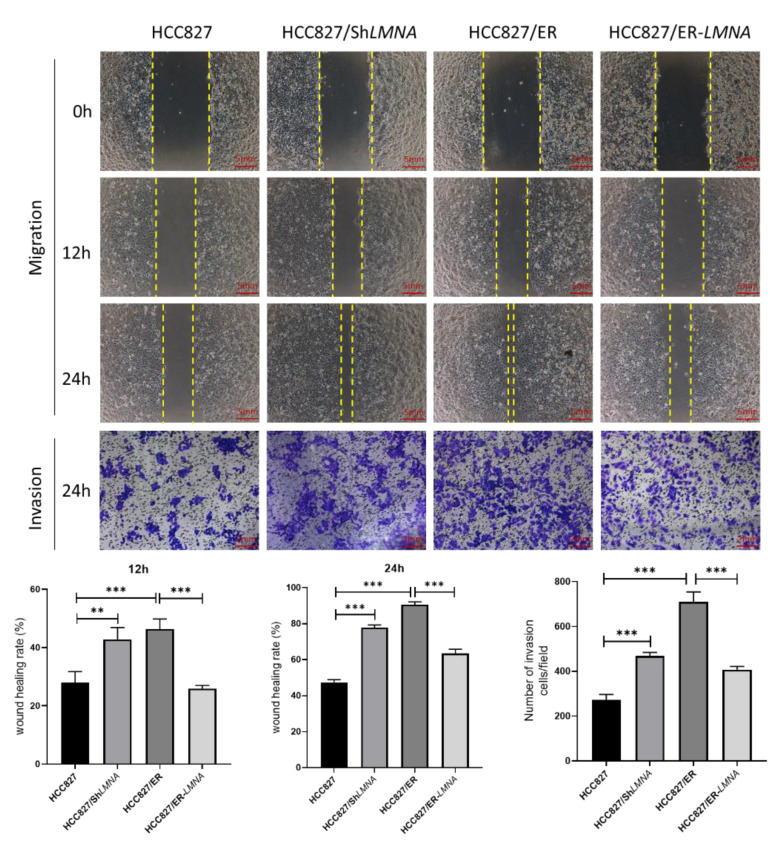
Effect of *LMNA* on the migration and invasive capabilities of HCC827 and HCC827/ER cells. Representative images show the migration and invasion ability of HCC827, HCC827/sh*LMNA*, HCC827/ER, and HCC827/ER-*LMNA* cells. Invasive cells were stained and counted under a microscope. Data are presented mean ± SD from five randomly selected visual fields. Significant differences are indicated as follows: Student’s *t*-test, ** *p* < 0.01, *** *p* < 0.001.

Malignant cells are well-known to be characterized by losing cell polarity and cell-cell adhesion while gaining migration and invasion capabilities [18]. To examine whether *LMNA* affected lung adenocarcinoma cell migration and invasion, wound-healing and transwell assays were performed. We observed that the ability to migrate and invade cells increased significantly in HCC827 cells after the elimination of *LMNA*. In contrast, wound-healing and transwell migration assays showed that *LMNA* overexpression dramatically inhibited the migration and invasion ability of HCC827/ER cells (Figure 4). In general, those results indicated that *LMNA* inhibited the migration and invasion of acquired erlotinib-resistant HCC827/ER cells.

### 2.5. LMNA Inhibition of EMT in Erlotinib-Resistant Lung Adenocarcinoma Cells

**Figure 5:**
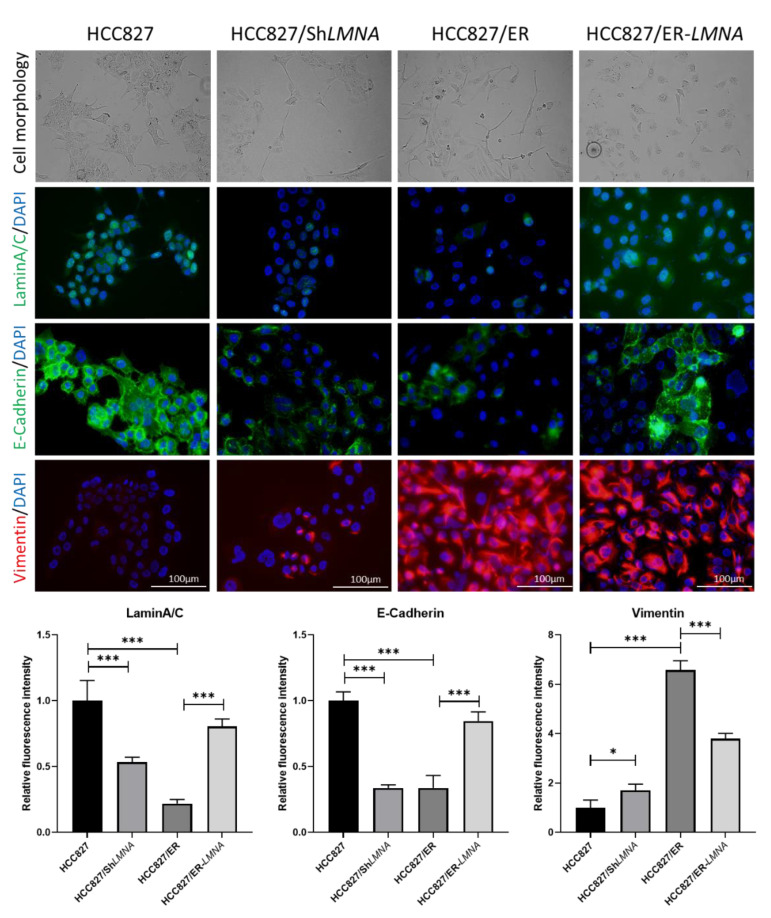
*LMNA* regulation of EMT in erlotinib-acquired resistant lung adenocarcinoma cells; morphological changes from an epithelial phenotype to a spindle-shaped EMT-like morphology in HCC827 cells after knockdown of *LMNA*. EMT-like morphology in HCC827/ER cells was reversed by overexpression of *LMNA*. Levels of laminA/C (green), E-cadherin (green), and vimentin (red) were determined by immunofluorescence assay. Nuclei were visualized using DAPI staining (blue). Data are presented mean ± SD from three independent experiments. Significant differences are indicated as follows: Student’s *t*-test, * *p* < 0.05, *** *p* < 0.001.

EMT is a hallmark of acquired resistance to EGFR-TKI and is associated with increased migration and invasion capacity of lung adenocarcinoma cells. To study the influence of *LMNA* on EMT in erlotinib-resistant HCC827/ER cells, the expression of EMT-related proteins was examined in HCC287, HCC287/sh*LMNA* (*LMNA* knockdown), HCC827/ER, and HCC287/ER-*LMNA* (*LMNA* overexpression) cells. As shown in Figure 5, HCC827 was in general high in E-cadherin and low in vimentin protein expression, but E-cadherin was significantly down-regulated and vimentin was significantly up-regulated in HCC827/ER cells. Transfection of *LMNA* target shRNA (HCC827/sh*LMNA*) cells showed a decrease in E-cadherin protein expression and an increase in vimentin protein expression. At the same time, overexpression of *LMNA* in HCC827/ER cells (HCC827/ER-*LMNA*) showed that E-cadherin was significantly up-regulated and vimentin was down-regulated. Furthermore, a clear change in cell morphology was observed in erlotinib-resistant cells as compared to parental cells. HCC827 cells displayed epithelial morphology, whereas HCC827/ER cells underwent EMT to form a fibroblast-like morphology. Furthermore, the elimination of *LMNA* in HCC827 cells displayed a spindle-shaped EMT-like morphology, and overexpression of *LMNA* reversed the EMT-like morphology in HCC827/ER cells. From these data, *LMNA* was involved in the phenotypic transformation of EMT to mediate the acquired resistance to erlotinib in NSCLC.

### 2.6. LMNA Regulation of Cytoskeletal Changes and Nuclear Deformability

**Figure 6:**
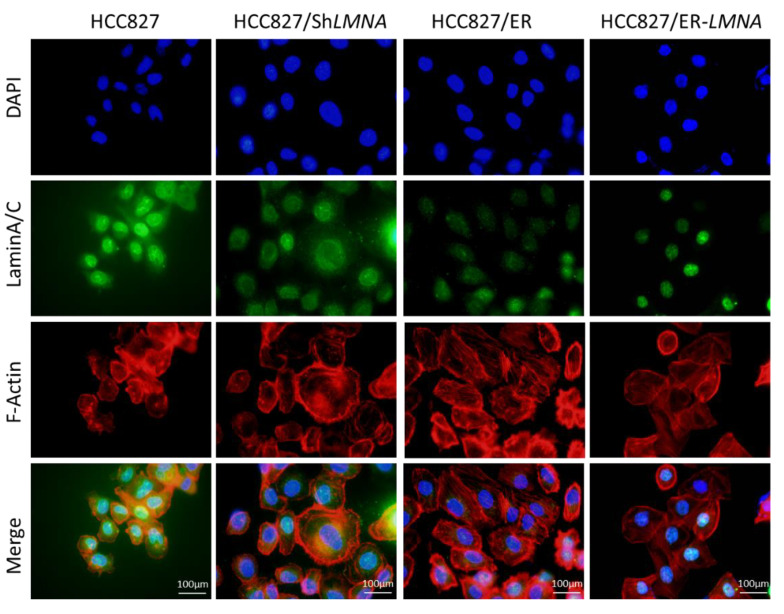
*LMNA* regulation of cytoskeletal changes and nuclear deformability. Cytoskeletal F-actin was stained with rhodamine phalloidin (red). Nuclei were visualized using DAPI staining (blue). LaminA/C was determined by immunofluorescence assay (green). Representative pictures of fluorescent laminA/C, F-actin, and nuclei are shown. Scale bar = 100 μm.

Due to the close correlation between nuclear lamina and the cytoskeleton, we evaluated whether *LMNA* impacted the shape and volume of the nucleus and cytoskeleton in erlotinib-resistant cells and parental cells. Therefore, the shape and volume of nuclear and cytoskeleton changes were detected in the four indicated cell lines by immunofluorescence. As shown in Figure 6, the nuclear size in HCC827/sh*LMNA* or HCC827/ER cells (low *LMNA* expression) was greater than that in HCC827 or HCC827/ER-*LMNA* cells (high *LMNA* expression). The results suggested that a decrease in *LMNA* expression resulted in a higher nuclear deformability. For cytoskeleton change, F-actin filaments were evaluated. We found a disrupted organization of F-actin filaments in low-*LMNA* expression cells, which was rescued by overexpression of *LMNA*. The F-actin filaments were loosely packed in HCC827/ER and HCC827/sh*LMNA* cells, resulting in resistant cells exhibiting a spindle-shaped EMT-like morphology. As expected, overexpression of *LMNA* promoted the reorganization of the F-actin filaments to reverse EMT-like morphology. Together, these results suggested that nuclear deformability and cytoskeletal changes depend on *LMNA* expression in NSCLC.

### 2.7. LMNA Reversal of EMT by Regulation of the FGFR/MAPK/c-fos Signaling Pathway

**Figure 7:**
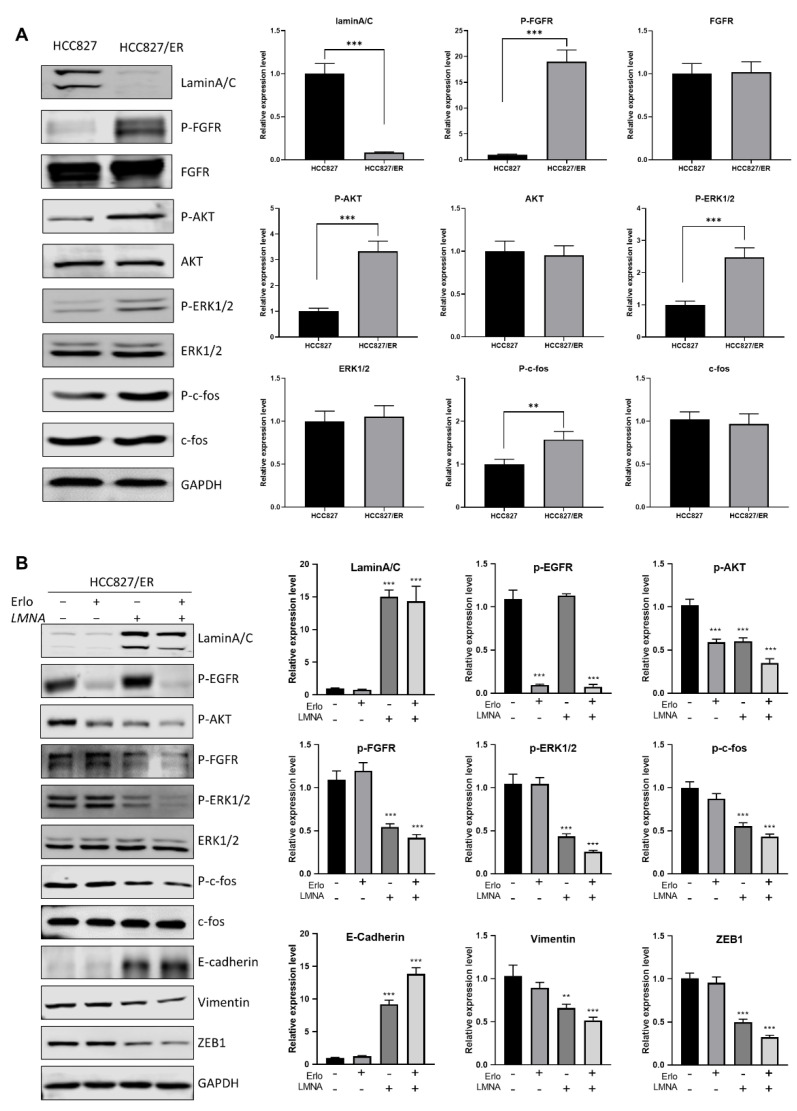
*LMNA* reversal of EMT by regulation of the FGFR/MAPK/c-fos signaling pathway. (**A**) Western blot analysis was performed to detect the expression of laminA/C, P-FGFR, P-AKT, P-ERK1/2, and P-c-fos in HCC827 and HCC827/ER cells. (**B**) HCC827/ER cells were transfected with *LMNA* expression vectors and treated with or without 2.5 μM erlotinib for 24 h, then subjected to Western blot analysis using the indicated primary antibody. Data are presented mean ± SD from three independent experiments. Significant differences are indicated as follows: Student’s *t*-test, ** *p* < 0.01, *** *p* < 0.001.

In order to address the molecular mechanism of *LMNA* in acquired resistance to erlotinib and regulation of migration, expression of several proteins of EMT was measured in erlotinib-resistant cells and parental cells. Western blot results showed that AKT(p-AKT), FGFR (p-FGFR), ERK1/2 (p-ERK1/2), and c-fos (p-c-fos) phosphorylation levels were up-regulated in HCC827/ER cells (low *LMNA* expression) as compared to HCC827 cells (Figure 7A). Similarly, the same tendency of p-AKT, p-FGFR, p-ERK1/2, and p-c-fos levels was maintained between HCC827 and HCC827/sh*LMNA* cells (Figure S1). These data demonstrated that AKT and FGFR/MAPK/c-fos signal pathways were activated to induce EMT and acquired resistance to erlotinib. Overexpression of *LMNA* in HCC827/ER cells reduced the phosphorylation of AKT, ERK1/2, c-fos, and FGFR. Furthermore, *LMNA* overexpression was synergized with 2.5 μM erlotinib treatment to induce lower phosphorylation of AKT, ERK1/2, c-fos, and FGFR compared to overexpression of *LMNA* alone in HCC827/ER cells (Figure 7B). As expected, overexpression of *LMNA* increased E-cadherin expression and decreased the expression of vimentin and ZEB1. This effect was enhanced by a combination of 2.5 μM erlotinib. Above all, *LMNA* overexpression inhibited cell proliferation of erlotinib-resistant cells by downregulation of AKT phosphorylation and also reversed EMT by downregulation of the FGFR/MAPK/c-fos pathways.

## 3. Discussion

For patients with NSCLC that harbors active EGFR mutations, it has been a first-line treatment to administer epidermal growth factor receptor tyrosine kinase inhibitors (EGFR-TKIs). However, it is true that the vast majority of patients who initially responded to EGFR-TKIs therapy eventually developed acquired resistance, which invariably limited the effectiveness of the therapy. EMT is a dynamic process that affects many malignancies, including drug resistance. Accumulating evidence has highlighted that EMT has long been associated with acquired resistance to EGFR-TKIs in NSCLC [19,20,21,22], but the mechanisms underlying the acquired resistance to EGFR-TKIs dependent on EMT remained poorly understood. In the present study, an erlotinib-resistant HCC827/ER cell line was established by treating EGFR-mutated HCC827 cells with increasing doses of erlotinib until resistance was achieved at 5 μM erlotinib. Consistently with previous reports, HCC827/ER cells exhibited an EMT-like phenotype [23]. Furthermore, we investigated the effect of *LMNA* on EMT to induce acquired resistance to erlotinib in NSCLC. To the best of our knowledge, this is the first time this has been reported.

The RNA sequencing (RNA-seq) technique is widely used to measure genomic transcript expression [24]. Therefore, we conducted RNA-seq and bioinformatics analysis to reveal multiple processes that were involved in acquired resistance to erlotinib. From the RNA-seq data, we found that resistant cells down-regulated the expression of epithelial marker *CDH1* while they up-regulated the expression of mesenchymal markers, such as *CDH2*, *VIM*, *SNAI2*, and *ZEB1*. In addition, EMT-related protein expression levels were consistent with gene expression levels in HCC827/ER cells by Western blot analysis. The above results further demonstrated that HCC827/ER cells displayed features of EMT, thus contributing to acquired resistance to erlotinib in NSCLC. Weng et al. also reported that NSCLC with acquired resistance to EGFR-TKI showed EMT characteristics, with a decrease in E-cadherin and an increase in vimentin without any secondary EGFR mutations [25]. Furthermore, the elimination of E-cadherin in parental cells increased EGFR-TKI resistance [26], while blocking the expression of vimentin in resistant cells resulted in opposite effects [27]. In this study, our results demonstrated that inhibition of EMT reversed acquired resistance of HCC827/ER cells to erlotinib.

It is well-known that laminA/C is in close contact with lamina-associated domains, which can help organize chromosomes inside the nucleus and play an important role in gene expression [28]. Grigoryan et al. demonstrated that laminA/C regulated nuclear shape and volume, and consequently, low levels of laminA/C conferred aging-associated phenotypic and functional changes in hematopoietic stem cells [12]. Similarly, our data demonstrated that *LMNA* (laminA/C) expression was down-regulated in erlotinib-resistant cells as compared to parental cells, and the nuclei of erlotinib-resistant cells displayed an increased nuclear volume. Interestingly, the level of laminA/C with acquired resistance to erlotinib was dramatically reduced and restored to the level of laminA/C found in resistant cells upon inhibition of nuclear shape and volume, further reversing resistance to erlotinib in NSCLC. This could indicate a molecular mechanism, involved in the laminA/C regulation structure of lamina-associated domains, which participates in nuclear architectural aspects and in genome maintenance by providing a scaffold to bind chromatin and protein complexes that regulate genomic stability [29]. Therefore, we further investigated whether laminA/C inhibited the EMT process by regulating nuclear volume and shape. As expected, a low level of laminA/C promoted the EMT process and induced acquired resistance to erlotinib, characterized by downregulation of E-cadherin and upregulation of N-cadherin, vimentin, snail, and ZEB1. On the contrary, overexpression of laminA/C in erlotinib-resistant cells reversed the EMT phenotype and restored cells’ sensitivity to erlotinib. Consistent with our results, Zuo et al. demonstrated that laminA/C overexpression promoted cells to acquire more epithelial phenotypes, and moreover, laminA/C knockdown induced cells to acquire more interstitial characteristics in prostate cancers [13]. Collectively, these experimental results revealed the notion that laminA/C plays a vital role in the development of acquired resistance to EGFR-TKIs.

**Figure 8:**
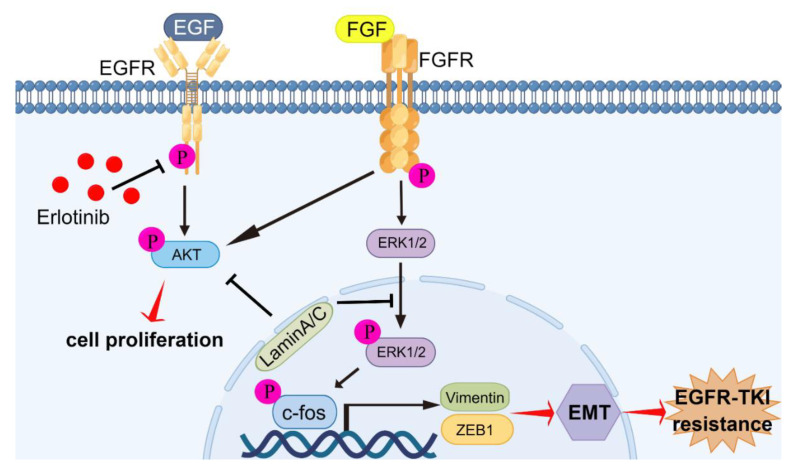
Graphic abstract of molecular mechanisms of laminA/C-regulated EMT promoting acquired resistance to erlotinib in NSCLC. LaminA/C inhibited the phosphorylation of AKT and FGFR to prevent cell proliferation of erlotinib-resistant cells. Meanwhile, laminA/C decreased ERK1/2 and c-fos activation and decreased vimentin and ZEB1 expressions, resulting in reversing of the process of EMT and improving of erlotinib sensitivity in NSCLC with EGFR mutation. By Figdraw (www.figdraw.com).

In previous studies, FGFR signaling has been associated with resistance to EGFR-TKIs caused by EMT [30,31,32]. By blocking both EGFR and FGFR, acquired resistance can be inhibited through blocking of the development of EMT in NSCLC with EGFR mutations [22,31]. In this study, we found that FGFR signaling was activated, resulting in acquired resistance of HCC827/ER cells and HCC827/sh*LMNA* cells to erlotinib. The level of EGFR phosphorylation was negatively regulated by treatment with erlotinib in HCC827/ER cells; however, cell proliferation was not inhibited. The reason for this was the compensatory mechanism, which also promoted cell proliferation and migration via the FGFR/MAPK/c-fos signaling pathway. Overexpression of *LMNA* inhibited phosphorylation levels of FGFR, AKT, ERK1/2, and c-fos. Furthermore, the combination of erlotinib and *LMNA* significantly enhanced the inhibitory effect on phosphorylation of FGFR, AKT, ERK1/2, and c-fos. The indicated c-fos was a transcription factor and regulated cell proliferation, differentiation, invasion, and metastasis [33]. A previous study demonstrated that c-fos bound to laminA/C and did not bind to related DNA sequences, but this was reversed by phosphorylating c-fos via the ERK1/2 pathway [8]. According to our data, erlotinib-resistant cells reduced laminA/C levels and decreased the binding of c-fos to laminA/C, leading to increased proliferation and migration. On the contrary, laminA/C overexpression inhibited FGFR signaling while increasing c-fos binding to laminA/C, resulting in blocking of the evolution of acquired resistance associated with EMT in NSCLC with EGFR mutations (Figure 8).

## 4. Materials and Methods

### 4.1. Cell Culture and Reagents

Erlotinib-resistant HCC827/ER cells were established as previously described [34] and cultured in a RPMI-1640 medium (Hyclone, Beijing, China, SH30809.01) supplemented with 10% FBS (R&S, Australia, 009106), penicillin and streptomycin (Gibco, New York*,* NY, USA, 15140122), and 5 μM erlotinib (Selleck, Shanghai, China, s1023) at 37 °C and 5% CO_2_. The parental HCC827 cell line was purchased from ATCC. The following antibodies were used: p-AKT^Ser473^ (Cell Signaling Technology, Danvers, MA, USA, 4060), AKT (Abcam, Shanghai, China, ab79360), E-cadherin (Proteintech, Wuhan, China, 20874-1-AP), N-cadherin (ZEN Bio, Chengdu, China, 382812), vimentin (ZEN Bio, Chengdu, China, R22775), snail (Cell Signaling Technology, 3879), laminA/C (Santa Cruz, Starr County, TX, USA, sc-376248), laminB1 (Cell Signaling Technology, 12586), ZEB1 (Cell Signaling Technology, 3396), p-FGFR (Abcam, Shanghai, China, ab192589), p-ERK1/2 (Cell Signaling Technology, 4370), ERK1/2 (Proteintech, Wuhan, China, 67170-1-Ig), p-c-fos (Santa Cruz, TX, USA, sc-81485), c-fos (ZEN Bio, Chengdu, China, 340249), p-EGFR^Y845^ (Cell Signaling Technology, 6963), and GAPDH (SAB, MD, USA, 48142).

### 4.2. RNA Sequencing Analysis

RNA sequencing (RNA-seq) and RNA-seq analysis were performed by HuaDa Gene Company (Shenzhen, China). Briefly, total RNA was isolated from HCC827 and HCC827/ER cells using Trizol (Invitrogen, Carlsbad, CA, USA, 15596026) and treated with DNase I (Invitrogen, 18047019). Quality and purity of RNA was first evaluated by an Agilent 2100 Bio Analyzer. Next, sequencing was performed using the BGISEQ-500 platform. DEGs between two samples were identified and subjected to hierarchical clustering, gene ontology (GO), and Kyoto Encyclopedia of Genes and Genomes (KEGG) enrichment analysis.

### 4.3. Quantitative Reverse Transcription (qRT)-PCR

To verify the DEG results, we used qRT-PCR. First-strand cDNA synthesis was performed using PrimeScript RT Master Mix (Takara, Shiga, Japan, RR036A). The indicated cDNA was diluted tenfold and used as a template for quantitative (q)PCR analysis using TB Green Premix Ex Taq II (Takara, RR820A). Relative levels of target gene were calculated using the 2^−ΔΔCt^ method and GAPDH mRNA levels were used for normalization. Gene-specific primer pairs are listed in Table S1.

### 4.4. Cell Transfection

The *LMNA* plasmid was purchased from Sino Biological (Beijing, China). The *LMNA* gene was then subcloned and inserted into a PLVX-puro lentivirus vector (Clontech, Shiga, Japan). The PLVX-shRNA1 lentivirus plasmid for *LMNA* shRNA was constructed as previously described [35]. The *LMNA* shRNA sequence was as follows: 5′-CTGACTTCCAGAAGAACA-3′. Lentivirus lacking the shRNA insert was used as a control.

To establish *LMNA* knockdown or overexpression of *LMNA* cells, 293T cells were transfected with the target plasmid along with psPAX2 and PMD2G packing plasmids with Lipo8000 Transfection Reagent (Beyotime, Shanghai, China, C0533). Next, 293T cell supernatants were collected and mixed with fresh medium to infect HCC827 or HCC827/ER cells along with 2 μg/mL polybrene (Beyotime, C0351). After 24 h, the supernatants were replaced with fresh medium containing puromycin (Beyotime, ST551) at a concentration of 5 μg/mL. The puomycin-resistant cells were isolated and used for further experiments.

### 4.5. Cell Proliferation Assay

HCC827, HCC827/sh*LMNA*, HCC827/ER, and HCC827/ER-*LMNA* cells were seeded in 96-well plates (5 × 10^3^ cells per well) and incubated for 24 h, then given treatment with erlotinib (0, 0.0005, 0.001, 0.005, 0.01, 0.05, 0.1, 0.5, 1, 5 μM) for 48 h. At the end of the incubation, 3-(4,5-dimethylthiazole)-2,5-diphenyltetrazolium bromide (MTT, Beyotime, Shanghai, China, ST316) was added into each well and incubated for an additional 4 h. Cell viability was determined through measuring of optical density at 490 nm using a Cytation 5 microplate reader (BioTek, Winooski, VT, USA).

### 4.6. Colony-Formation Assay

HCC827, HCC827/sh*LMNA*, HCC827/ER, and HCC827/ER-*LMNA* cells were seeded in a 6-well dish at a density of 1000 cells/well. Cells were treated with erlotinib at 5 nM or 2.5 μM for 14 days to allow colony formation. Colonies were fixed and stained with 0.1% crystal violet staining solution at room temperature, and were counted and photographed by a scanner (Epson, China). Three independent experiments were carried out.

### 4.7. Wound Healing Assay

HCC827, HCC827/sh*LMNA*, HCC827/ER, and HCC827/ER-*LMNA* cells were seeded in a 12-well plate and a yellow pipe was used to wound the cells. Wounded cells were lightly washed with phosphate-buffered saline (PBS) prior to receiving the indicated treatment. After incubation for 12 or 24 h, the migrated distances of different group cells were analyzed by Olympus IX53 microscope (Olympus, Tokyo, Japan) with five randomly chosen fields.

### 4.8. Transwell Assay

The cell migration assay was performed using a 8 μm Transwell Boyden chamber (Corning, Tewksbury, MA, USA, 3422) with Matrigel (Corning, 356231). Cells were suspended in serum-free RPMI-1640 and approximately 5 × 10^4^ cells were added into the upper chamber and placed in a 24-well plate containing a medium with 10% FBS. After 24 h of incubation, the Matrigel was gently removed and the cells were fixed with methanol. Subsequently, 0.1% crystal violet was used to stain the cells. Lastly, the cells were observed under Olympus IX53 microscope (Olympus) and five randomly chosen fields were analyzed for each group.

### 4.9. Immunofluorescence

Cells were seeded onto cover glasses in a 12-well plate and fixed with 4% PFA for 30 min, followed by permeabilization with 0.1% Triton-X100 for 15 min. After blocking with 3% bovine serum albumin for 1 h, the cover glasses were incubated overnight at 4 °C with primary antibodies. The cover glasses were incubated for 2 h with Alexa Fluor conjugated secondary antibodies and then stained for 20 min with DAPI. The protein expression or cytoskeleton in cells was photographed using fluorescence microscopy (Olympus).

### 4.10. Western Blot

Protein samples from the indicated cells were prepared in a RIPA buffer (Beyotime, Shanghai, China, P0013C) with a cocktail inhibitor (Roche, Germany, 11836170001) and boiled for 5 min. 20 μg of each sample was loaded into SDS-polyacrylamide gel. After SDS-PAGE, the proteins in the gel were transferred onto a polyvinylidene fluoride (PVDF) membrane (Millipore, Germany, IPVH00010). The membrane was then blocked in 5% fat-free milk for 2 h at room temperature and then incubated with primary antibodies overnight at 4 °C. The corresponding secondary antibodies were incubated for 1 h before protein detection using an Odyssey Imaging System (Li-COR, Lincoln, NE, USA).

### 4.11. Statistical Analysis

All data were expressed as mean ± standard error. All statistical analyzes were performed with GraphPad Prism 9.0.0 (GraphPad Software Inc., San Diego, CA, USA). Comparisons between two groups were made with Student’s *t*-test. *p* < 0.05 was considered statistically significant.

## Data Availability

Not applicable.

## Supplementary Materials

The supporting information can be downloaded at https://www.mdpi.com/article/10.3390/ijms232113237/s1.

## References

[1] Gentles A.J., Hui A.B., Feng W., Azizi A., Nair R.V., Bouchard G., Knowles D.A., Yu A., Jeong Y., Bejnood A. (2020). A human lung tumor microenvironment interactome identifies clinically relevant cell-type cross-talk. Genome Biol..

[2] Maron S.B., Alpert L., Kwak H.A., Lomnicki S., Chase L., Xu D., O’Day E., Nagy R.J., Lanman R.B., Cecchi F. (2018). Targeted Therapies for Targeted Populations: Anti-EGFR Treatment for EGFR-Amplified Gastroesophageal Adenocarcinoma. Cancer Discov..

[3] Janjigian Y.Y., Smit E.F., Groen H.J., Horn L., Gettinger S., Camidge D.R., Riely G.J., Wang B., Fu Y., Chand V.K. (2014). Dual inhibition of EGFR with afatinib and cetuximab in kinase inhibitor-resistant EGFR-mutant lung cancer with and without T790M mutations. Cancer Discov..

[4] Koulouris A., Tsagkaris C., Corriero A.C., Metro G., Mountzios G. (2022). Resistance to TKIs in EGFR-Mutated Non-Small Cell Lung Cancer: From Mechanisms to New Therapeutic Strategies. Cancers.

[5] Khaddour K., Jonna S., Deneka A., Patel J.D., Abazeed M.E., Golemis E., Borghaei H., Boumber Y. (2021). Targeting the Epidermal Growth Factor Receptor in EGFR-Mutated Lung Cancer: Current and Emerging Therapies. Cancers.

[6] Worman H.J. (2012). Nuclear lamins and laminopathies. J. Pathol..

[7] Iyer K.V., Taubenberger A., Zeidan S.A., Dye N.A., Eaton S., Julicher F. (2021). Apico-basal cell compression regulates Lamin A/C levels in epithelial tissues. Nat. Commun..

[8] Ding Y., Hao K., Li Z., Ma R., Zhou Y., Zhou Z., Wei M., Liao Y., Dai Y., Yang Y. (2020). c-Fos separation from Lamin A/C by GDF15 promotes colon cancer invasion and metastasis in inflammatory microenvironment. J. Cell. Physiol..

[9] Kaspi E., Frankel D., Guinde J., Perrin S., Laroumagne S., Robaglia-Schlupp A., Ostacolo K., Harhouri K., Tazi-Mezalek R., Micallef J. (2017). Low lamin A expression in lung adenocarcinoma cells from pleural effusions is a pejorative factor associated with high number of metastatic sites and poor Performance status. PLoS ONE.

[10] Maresca G., Natoli M., Nardella M., Arisi I., Trisciuoglio D., Desideri M., Brandi R., D’Aguanno S., Nicotra M.R., D’Onofrio M. (2012). LMNA knock-down affects differentiation and progression of human neuroblastoma cells. PLoS ONE.

[11] Nardella M., Guglielmi L., Musa C., Iannetti I., Maresca G., Amendola D., Porru M., Carico E., Sessa G., Camerlingo R. (2015). Down-regulation of the Lamin A/C in neuroblastoma triggers the expansion of tumor initiating cells. Oncotarget.

[12] Grigoryan A., Guidi N., Senger K., Liehr T., Soller K., Marka G., Vollmer A., Markaki Y., Leonhardt H., Buske C. (2018). LaminA/C regulates epigenetic and chromatin architecture changes upon aging of hematopoietic stem cells. Genome Biol..

[13] Zuo L., Zhao H., Yang R., Wang L., Ma H., Xu X., Zhou P., Kong L. (2018). Lamin A/C might be involved in the EMT signalling pathway. Gene.

[14] Zhang X., Lv Y. (2017). Suspension state increases reattachment of breast cancer cells by up-regulating lamin A/C. Biochim. Et Biophys. Acta. Mol. Cell Res..

[15] Fujiwara C., Muramatsu Y., Nishii M., Tokunaka K., Tahara H., Ueno M., Yamori T., Sugimoto Y., Seimiya H. (2018). Cell-based chemical fingerprinting identifies telomeres and lamin A as modifiers of DNA damage response in cancer cells. Sci. Rep..

[16] Zhang Y., Wang J., Huang W., Cai J., Ba J., Wang Y., Ke Q., Huang Y., Liu X., Qiu Y. (2018). Nuclear Nestin deficiency drives tumor senescence via lamin A/C-dependent nuclear deformation. Nat. Commun..

[17] Matralis A.N., Xanthopoulos D., Huot G., Lopes-Paciencia S., Cole C., de Vries H., Ferbeyre G., Tsantrizos Y.S. (2018). Molecular tools that block maturation of the nuclear lamin A and decelerate cancer cell migration. Bioorg. Med. Chem..

[18] Nguyen B.T., Pyun J.C., Lee S.G., Kang M.J. (2019). Identification of new binding proteins of focal adhesion kinase using immunoprecipitation and mass spectrometry. Sci. Rep..

[19] Zhu X., Chen L., Liu L., Niu X. (2019). EMT-Mediated Acquired EGFR-TKI Resistance in NSCLC: Mechanisms and Strategies. Front. Oncol..

[20] Chen H., Yang D., Wang Y., Tao H., Luo Y., Wu A., Li S., Yang Z., Chen M. (2022). Activation of the Hedgehog pathway mediates resistance to epidermal growth factor receptor inhibitors in non-small cell lung cancer. J. Cancer.

[21] Wang C.Y., Lee M.H., Kao Y.R., Hsiao S.H., Hong S.Y., Wu C.W. (2021). Alisertib inhibits migration and invasion of EGFR-TKI resistant cells by partially reversing the epithelial-mesenchymal transition. Biochim. Et Biophys. Acta Mol. Cell Res..

[22] Clement M.S., Gammelgaard K.R., Nielsen A.L., Sorensen B.S. (2020). Epithelial-to-mesenchymal transition is a resistance mechanism to sequential MET-TKI treatment of MET-amplified EGFR-TKI resistant non-small cell lung cancer cells. Transl. Lung Cancer Res..

[23] Li M., Chen H., Sun T., Ma Z., Chen X., Wu D., Huang W., Wang X. (2020). p70S6K Promotes Acquired Resistance of Erlotinib Through Induction of Epithelial-Mesenchymal Transition in Non-Small Cell Lung Carcinoma. OncoTargets Ther..

[24] Hedges D.J. (2022). RNA-seq Fusion Detection in Clinical Oncology. Adv. Exp. Med. Biol..

[25] Weng C.H., Chen L.Y., Lin Y.C., Shih J.Y., Lin Y.C., Tseng R.Y., Chiu A.C., Yeh Y.H., Liu C., Lin Y.T. (2019). Epithelial-mesenchymal transition (EMT) beyond EGFR mutations per se is a common mechanism for acquired resistance to EGFR TKI. Oncogene.

[26] Lin S., Zhang R., An X., Li Z., Fang C., Pan B., Chen W., Xu G., Han W. (2019). LncRNA HOXA-AS3 confers cisplatin resistance by interacting with HOXA3 in non-small-cell lung carcinoma cells. Oncogenesis.

[27] Xue S., Wu W., Wang Z., Lu G., Sun J., Jin X., Xie L., Wang X., Tan C., Wang Z. (2020). USP5 Promotes Metastasis in Non-Small Cell Lung Cancer by Inducing Epithelial-Mesenchymal Transition via Wnt/beta-Catenin Pathway. Front. Pharmacol..

[28] van Steensel B., Belmont A.S. (2017). Lamina-Associated Domains: Links with Chromosome Architecture, Heterochromatin, and Gene Repression. Cell.

[29] Liu B., Wang J., Chan K.M., Tjia W.M., Deng W., Guan X., Huang J.D., Li K.M., Chau P.Y., Chen D.J. (2005). Genomic instability in laminopathy-based premature aging. Nat. Med..

[30] Ware K.E., Hinz T.K., Kleczko E., Singleton K.R., Marek L.A., Helfrich B.A., Cummings C.T., Graham D.K., Astling D., Tan A.C. (2013). A mechanism of resistance to gefitinib mediated by cellular reprogramming and the acquisition of an FGF2-FGFR1 autocrine growth loop. Oncogenesis.

[31] Raoof S., Mulford I.J., Frisco-Cabanos H., Nangia V., Timonina D., Labrot E., Hafeez N., Bilton S.J., Drier Y., Ji F. (2019). Targeting FGFR overcomes EMT-mediated resistance in EGFR mutant non-small cell lung cancer. Oncogene.

[32] Yu T., Xia Q., Gong T., Wang J., Zhong D. (2020). Molecular mechanism of acquired drug resistance in the EGFR-TKI resistant cell line HCC827-TR. Thorac. Cancer.

[33] Atsaves V., Leventaki V., Rassidakis G.Z., Claret F.X. (2019). AP-1 Transcription Factors as Regulators of Immune Responses in Cancer. Cancers.

[34] Nilsson M.B., Sun H., Diao L., Tong P., Liu D., Li L., Fan Y., Poteete A., Lim S.O., Howells K. (2017). Stress hormones promote EGFR inhibitor resistance in NSCLC: Implications for combinations with beta-blockers. Sci. Transl. Med..

[35] Bertero A., Pawlowski M., Ortmann D., Snijders K., Yiangou L., Cardoso de Brito M., Brown S., Bernard W.G., Cooper J.D., Giacomelli E. (2016). Optimized inducible shRNA and CRISPR/Cas9 platforms for in vitro studies of human development using hPSCs. Development.

