# Supplementary material for: LMNA Reduced Acquired Resistance to Erlotinib in NSCLC by Reversing the Epithelial–Mesenchymal Transition via the FGFR/MAPK/c-fos Signaling Pathway
Source: Int J Mol Sci. 2022 Oct 31;23(21):13237. doi: 10.3390/ijms232113237 (PMC9658955; doi:10.3390/ijms232113237)
Supplement: Supplementary file 1 [file ijms-23-13237-s001.zip › ijms-1978673-supplementary.pdf]

Table S1. List of primers for qRT-PCR

Gene name	Primer sequences (5’-3’)
LMNA	FW: ATGAGGACCAGGTGGAGCAGTA
	RV: ACCAGGTTGCTGTTCCTCTCAG
LMNB1	FW: GAGAGCAACATGATGCCCAAGTG
	RV: GTTCTTCCCTGGCACTGTTGAC
CDH1	FW: GCCTCCTGAAAAGAGAGTGGAAG
	RV: TGGCAGTGTCTCTCCAAATCCG
CDH2	FW: CCTCCAGAGTTTACTGCCATGAC
	RV: GTAGGATCTCCGCCACTGATTC
VIM	FW: AGGCAAAGCAGGAGTCCACTGA
	RV: ATCTGGCGTTCCAGGGACTCAT
SNAI1	FW: TGCCCTCAAGATGCACATCCGA
	RV: GGGACAGGAGAAGGGCTTCTC
TWIST1	FW: GCCAGGTACATCGACTTCCTCT
	RV: TCCATCCTCCAGACCGAGAAGG
ZEB1	FW: GGCATACACCTACTCAACTACGG
	RV: TGGGCGGTGTAGAATCAGAGTC
GAPDH	FW: GTCTCCTCTGACTTCAACAGCG
	RV: ACCACCCTGTTGCTGTAGCCAA

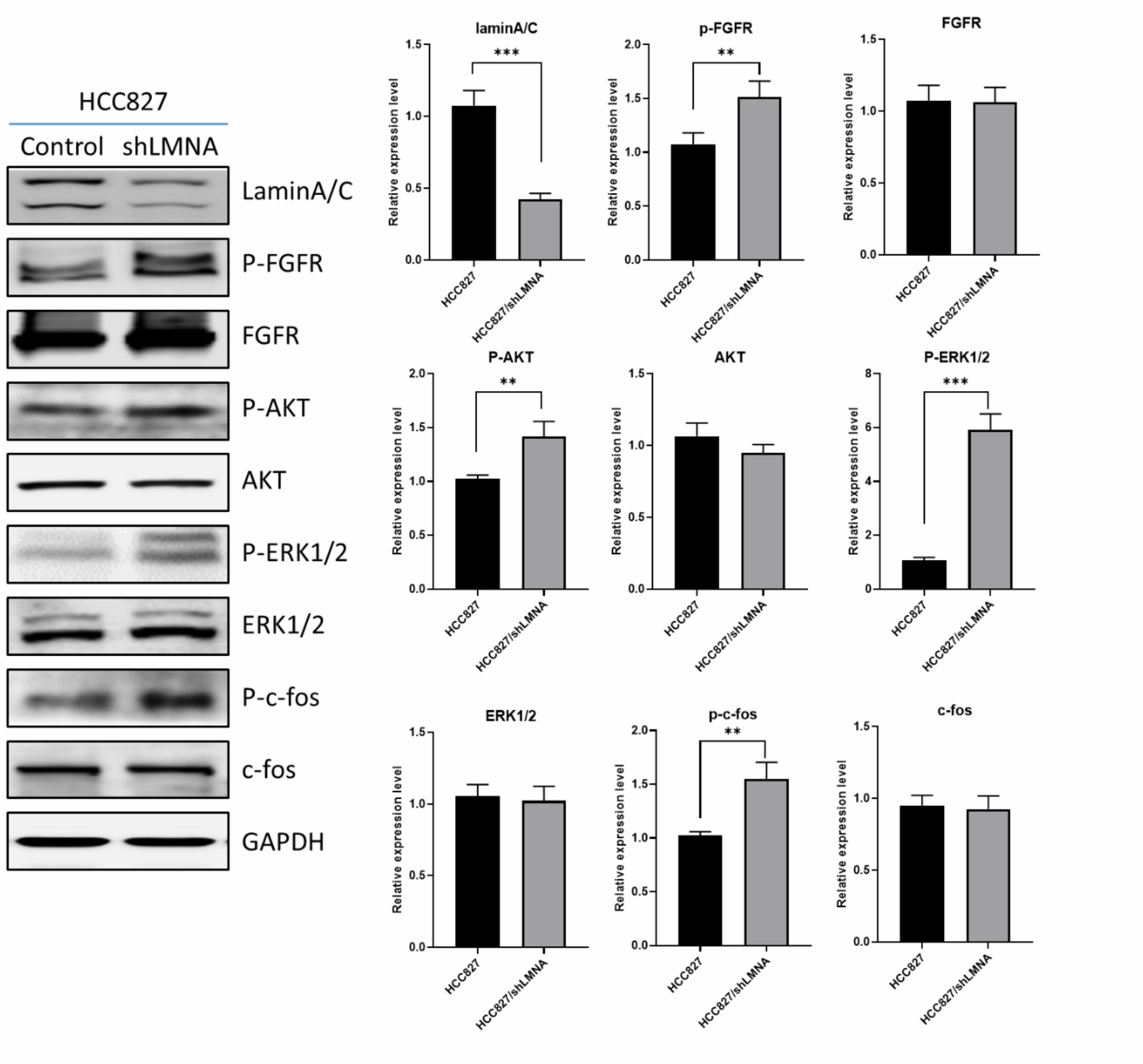

Figure S1. Knockdown of LMNA activated the FGFR/MAPK/c-fos signaling pathway in HCC827 cells. Western blot analysis was performed to detect the expression of laminA/C, P-FGFR, P-AKT, P-ERK1/2, P-c-fos in HCC827 and HCC827/shLMNA cells. Data are presented mean±SD from three independent experiments. Significant differences are indicated as follows: Student's t-test, ** $p < 0.01$, *** $p < 0.001$.
